# Characteristics of Canadians who use vaping products, by smoking status: findings from the Canadian Community Health Survey, 2020

**DOI:** 10.24095/hpcdp.44.11/12.02

**Published:** 2024-11

**Authors:** Christine D. Czoli, Camille Guertin, Daniel Dubois, Nancy Farrell, Gabriella Luongo, Gillian Williams, Trevor Mischki

**Affiliations:** 1 Tobacco Control Directorate, Controlled Substances and Cannabis Branch, Health Canada, Ottawa, Ontario, Canada

**Keywords:** electronic nicotine delivery systems, vaping, cigarette smoking, public health

## Abstract

**Introduction:**

To date, surveillance of vaping among Canadians (using vaping products with or without nicotine) has largely been examined with respect to age and smoking status. However, a nationally representative examination of a broad set of characteristics is lacking. This study characterized Canadians aged 15 years and older who vape, stratified by smoking status.

**Methods:**

Data from the 2020 Canadian Community Health Survey (unweighted analytical sample size: 28413 respondents) were used to examine past-30-day vaping stratified by smoking status (current smoking, former smoking, and never/nonsmoking). A Sex- and Gender-Based Analysis Plus approach was used to select individual-level characteristics for analysis. Descriptive statistics were used to examine outcomes by each characteristic and multivariable logistic regression models were constructed to identify significant factors associated with each past-30-day vaping by smoking status category, using weighted data.

**Results:**

In 2020, 2.0% (605000) of Canadians aged 15 years and older reported vaping and current smoking (dual use), 1.2% (372000) reported vaping and former smoking and 1.1% (352000) reported vaping and never/nonsmoking. Within each past-30-day vaping by smoking status category, certain subgroups presented higher risks: youth and young adults, men, and those having a mood and/or anxiety disorder had higher odds of dual use. Vaping and former smoking was associated with self-identification as a man, having a mood and/or anxiety disorder and provincial region. Youth and young adults, men and those identifying as not a visible minority had higher odds of vaping and never/nonsmoking.

**Conclusion:**

This analysis of Canadians who vape, stratified by smoking status, identifies high-prevalence subpopulations and informs us of the composition of vaping populations by select characteristics, deepening our understanding of Canadians who engage in vaping behaviours.

**Highlights**

- We used a Sex- and Gender-Based Analysis Plus approach in this study to characterize Canadians who use vaping products, as a function of smoking status.
- Vaping and current smoking (dual use) was associated with young age, identification as a man and mental health disorders. Vaping and former smoking was associated with identification as a man, mental health disorders and provincial region. Vaping and never/ nonsmoking was associated with young age and identification as a man and not belonging to a visible minority.
- Findings shed light on the composition of subpopulations that engage in vaping, which may inform equity considerations and research on interventions and public communications.

## Introduction

In recent years, the use of vaping products (with or without nicotine) has increased substantially among Canadians, particularly youth. Vaping products are battery-operated devices that heat a liquid solution, usually containing nicotine and flavours, but not tobacco. The Canadian Student Tobacco, Alcohol and Drugs Survey (CSTADS) showed an approximate doubling of the prevalence of vaping among Canadian students, from 10% in 2016/17 to 20% in 2018/19, which remained stable through 2021/22.^1^

Data from the Canadian Tobacco and Nicotine Survey (CTNS) similarly reflect this stabilization of vaping among youth aged 15 to 19 years between 2019 and 2022; in contrast, vaping among young adults aged 20 to 24 years increased between 2020 and 2022, while vaping among adults aged 25 and older remained stable from 2019 to 2022.^2^ Evidence also reveals a central role of cigarette smoking status: it is a robust and consistent correlate of vaping,^3^-^6^ and most Canadians aged 15 years and older who vape report currently or formerly smoking, although this also varies by age group.^2^ Thus, age and smoking status have been critical to understanding the emergence of vaping in Canada to date. 

These characteristics are also relevant to research on public health policy. Emerging evidence over the last decade reflects the challenge posed by vaping products: they present potential benefits as a smoking cessation tool to the millions of Canadians who smoke cigarettes, yet potential harms to individuals, particularly youth, who use the products but do not smoke.^7^ The impact of vaping products on individuals who formerly smoked cigarettes—with respect to whether these products encourage or deter relapse to smoking—remains unclear.^7^ Canada’s Tobacco Strategy aims to provide people who smoke access to less harmful sources of nicotine, while protecting youth and nonusers of tobacco products from nicotine addiction.8 In essence, rather than treating Canadians who vape as a homogeneous group, the Strategy considers the interplay of vaping and smoking status, recognizing that reasons for use, patterns of product use and, ultimately, associated public health impacts, will likely differ depending on the smoking status of the individuals who use them. 

Tobacco control research has demonstrated that many factors are relevant to understanding the epidemiology of cigarette smoking. In Canada, smoking prevalence over time has varied by sex,^9^ and disparities in smoking have been observed by household income and mental health.^10^ However, it is less clear whether there are characteristics, other than age and smoking status, relevant to the epidemiology of vaping. To date, national surveillance of vaping among Canadian youth and adults has been limited, often assessing prevalence by age group or grade, sex or gender, and smoking status.^1^,^2^,^11^ Several studies have assessed additional characteristics, including ethnicity, province of residence, household income and perceived physical and mental health, although these have been limited to subpopulations, including Canadian students^4^,^6^ and Canadians aged 15 years and older living in Ontario and Quebec.^5^

Additional studies have examined vaping among subpopulations of Canadians, examining various individual-, interpersonal- and environmental-level characteristics;^12^-^15^ however, these are based on convenience samples, meaning the results have limited generalizability. Thus, a nationally representative examination of a broad set of characteristics is lacking. To address this evidence gap, this study aimed to characterize Canadians who use vaping products, stratified by smoking status. 

## Methods

***Data source and study population***

The Canadian Community Health Survey (CCHS) is a cross-sectional survey administered by Statistics Canada that collects information related to health status, health care utilization and health determinants of Canadians annually (January to December each year).^16^ The survey covers approximately 98% of the Canadian population aged 12 years and older. Excluded from the sampling frame are individuals living on reserves and Crown lands in the provinces, institutional residents, full-time members of the Canadian Forces, youth aged 12 to 17 living in foster homes and residents of certain remote regions.^16^

Data were sourced from the 2020 CCHS Rapid Response file for examination of vaping using the Tobacco Alternatives and Vaping (TAV) module, given this was the first cycle of CCHS that reported on the use of vaping products across all Canadian provinces.^17^ Access to the data was provided by Health Canada’s Health Care Strategies Directorate. Ethical approval for population surveys conducted by Statistics Canada is based on the authority of the *Statistics Act* of Canada.

***Data analysis***

Key outcomes (there were 3) were past-30-day vaping stratified by smoking status—current smoking, former smoking and never/nonsmoking—to align with the aims of Canada’s Tobacco Strategy (Table 1). 

**Table 1:** Overview of study measures, Canadian Community Health Survey 2020

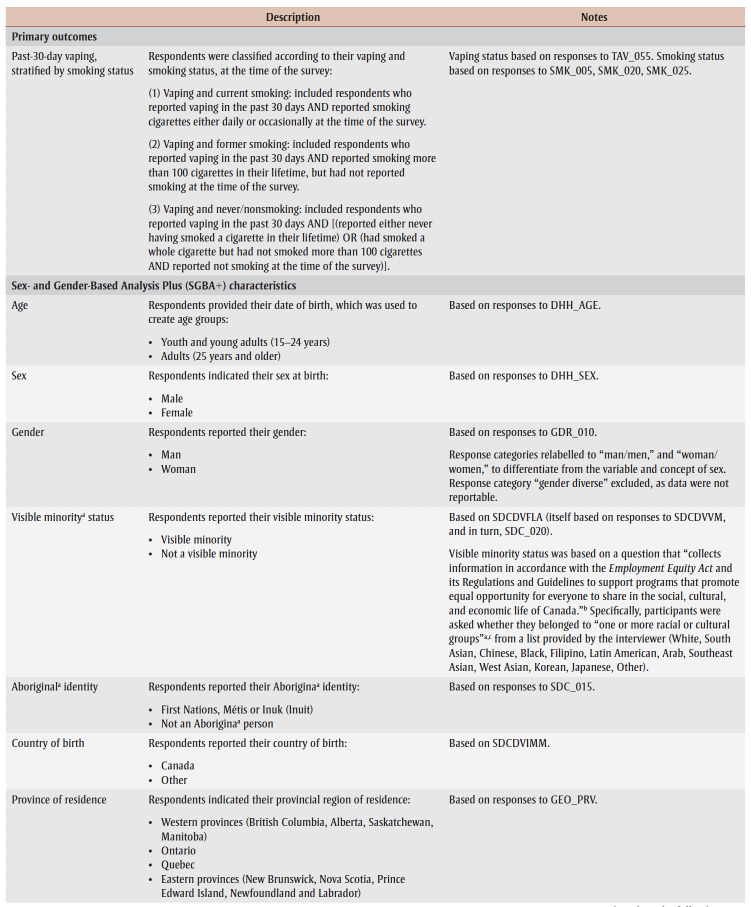 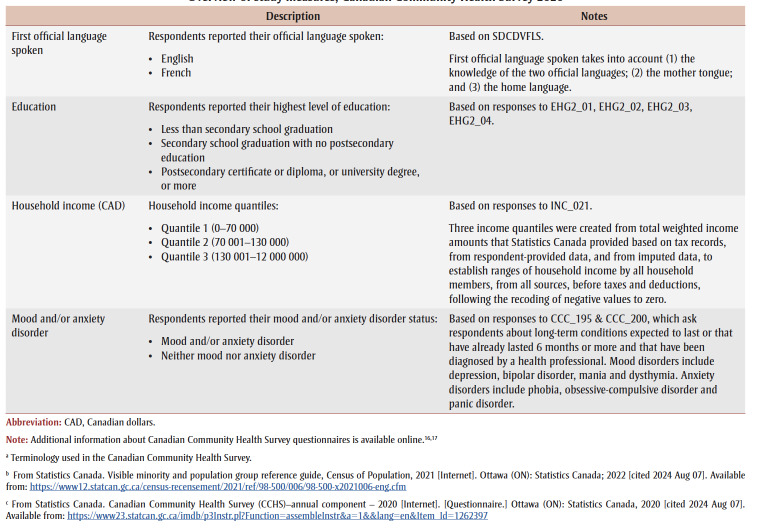

Selection of individual-level characteristics was guided by a Sex- and Gender-Based Analysis Plus (SGBA+) approach, which is an intersectional approach to assessing how a range of factors impacts individuals’ lived realities and differences in health outcomes,^18^ as well as by data availability. The final set of characteristics included age, sex, gender, country of birth, province, first official language, visible minority* status, Aboriginal* (Indigenous) identity, education, household income and mood and/or anxiety disorder status (Table 1). Results are presented below in accordance with Statistics Canada release guidelines. 

Analyses were conducted using weighted data. Statistics Canada survey sampling weights using the bootstrap method (1000 replicates) were applied to estimate standard error and account for the complex survey design. The analysis was limited to respondents aged 15 years and older to better align results with other surveillance tools used by Health Canada. Respondents with missing data for key outcomes (less than 0.5%) were excluded from the analysis, yielding an unweighted analytical sample size of 28 413 (n=399 for vaping and current smoking, n=309 for vaping and former smoking and n=260 for vaping and never/nonsmoking; n=27 445 for not vaping). 

Descriptive statistics were generated to estimate the weighted prevalence of key outcomes across levels of SGBA+ characteristics, using Pearson chi-square tests with Rao-Scott correction to denote differences (e.g. for the outcome of vaping and current smoking by gender: prevalence of Canadian women and Canadian men who reported vaping and current smoking). We also described the proportion of each level of the SGBA+ characteristics among Canadians who reported each key outcome (e.g. for the outcome of vaping and current smoking by gender: proportion of Canadians who reported vaping and current smoking who identified as women and who identified as men). 

Multivariable logistic regression models were estimated and assessed using the approach by Zhang^19^ to examine correlates of each key outcome; a description of the final model for each key outcome is included alongside the results, in the next section. Given the extent to which sex and gender are correlated, we chose to include gender in our model-building exercise because socially constructed roles, behaviours, expressions and identities may be more relevant to shaping behaviours with respect to tobacco and vaping products than physiological sex differences.^18^ Analyses were conducted using Stata version 17.0 (StataCorp LP, College Station, TX, US) with a *p* value set at <0.05 to denote statistical significance. 

*Terminology used in the Canadian Community Health Survey.

## Results

In 2020, 2.0% (605 000) of Canadians reported vaping and current smoking (dual use), 1.2% (372 000) reported vaping and formerly smoking and 1.1% (352 000) reported vaping and never/nonsmoking (Table 2). 

**Table 2:** Characterization of Canadians aged 15 years and older who reported past-30-day vaping, by smoking status, 2020

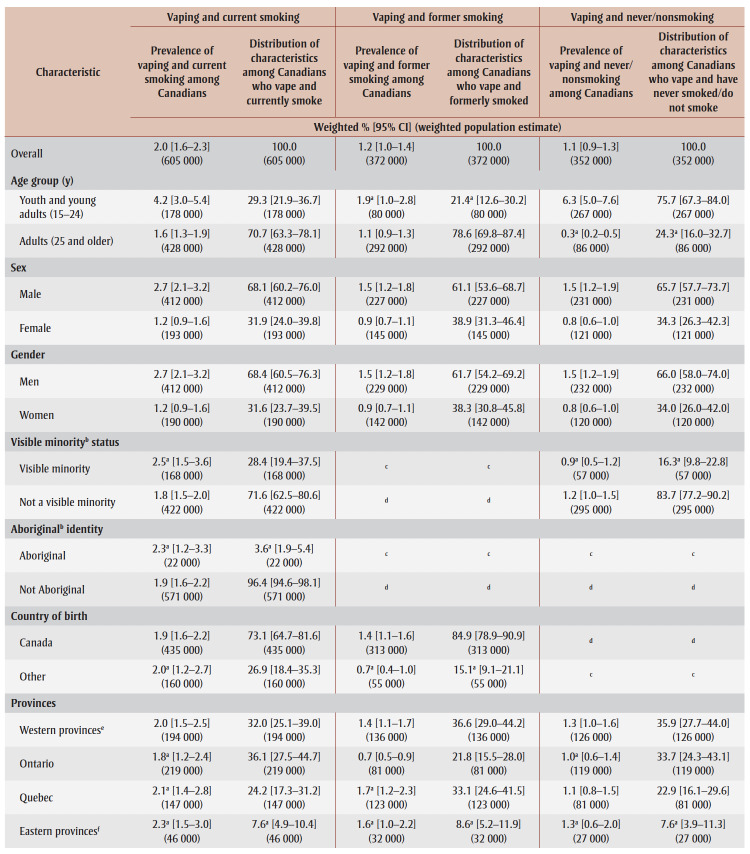 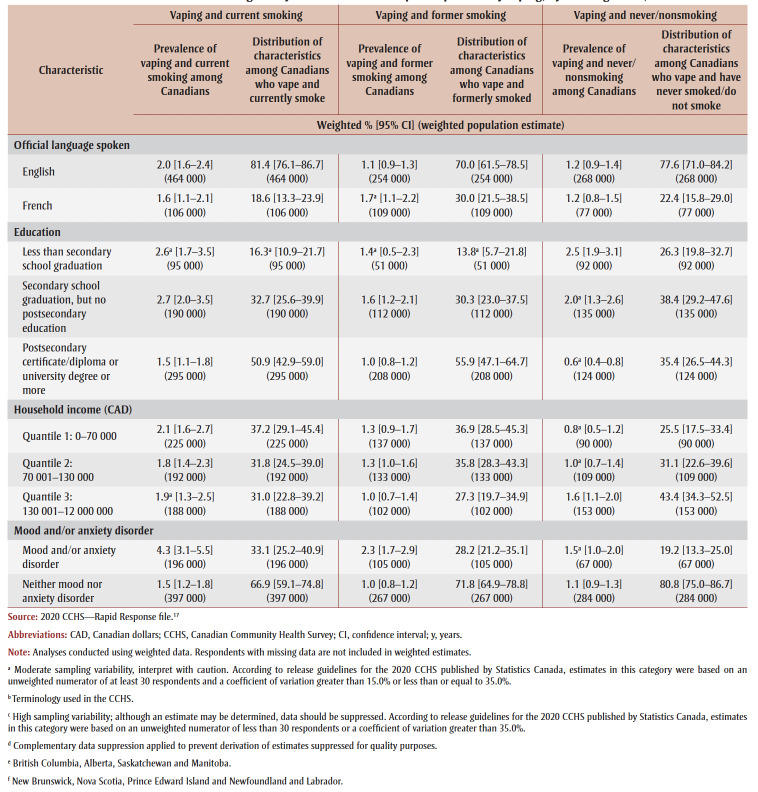

***Vaping and current smoking (dual use)***

As shown in Table 2, vaping and current smoking (i.e. dual use) varied significantly by age (*p*<0.001). Prevalence of dual use was significantly higher among youth and young adults aged 15 to 24years (4.2%) compared to adults aged 25 years and older (1.6%); however, adults aged 25 years and older made up the majority (70.7%) of Canadians who reported dual use. 

Dual use varied significantly by sex at birth (*p*<0.001) and self-reported gender identity (*p*<0.001). Prevalence of dual use was significantly higher among males (2.7%) and men (2.7%), when compared to females (1.2%) and women (1.2%). Most Canadians who reported dual use were male (68.1%, vs. female, 31.9%) and identified as men (68.4%, vs. women, 31.6%). 

Dual use did not vary by country of birth (*p*=0.90), visible minority status (*p*=0.15) or Aboriginal identity (*p*=0.56). Most Canadians who reported dual use were born in Canada (73.1%) and identified neither as a visible minority (71.6%) nor as Aboriginal (96.4%). 

Dual use did not vary by provincial region (*p*=0.78). Most Canadians who reported dual use lived in Ontario (36.1%) or the western provinces (32.0%). 

Dual use also did not vary by official language (*p*=0.21); however, most Canadians who reported dual use indicated English as their first official language spoken (81.4%). 

Dual use varied significantly by education (*p*<0.01). Canadians with less than secondary school graduation (2.6%†) and with secondary school graduation (2.7%) had significantly higher prevalence rates of dual use, compared to those with a postsecondary certificate/diploma or university degree or more (1.5%). Approximately half (50.9%) of Canadians who reported dual use had completed postsecondary education, while about one-third (32.7%) had completed secondary school, and the remainder (16.3%†) had less than secondary school education. 

Dual use did not vary by household income (*p*=0.71). Among Canadians who reported dual use, household income was relatively evenly distributed. 

Dual use varied significantly by mental health status (*p*<0.001). Canadians with a mood and/or anxiety disorder had a higher prevalence rate (4.3%) than Canadians without these disorders (1.5%); however, most Canadians reporting dual use had neither a mood nor an anxiety disorder (66.9%). 

The final multivariable model examining vaping and current smoking was fitted with age group, gender and mental health. Results showed higher odds of dual use among youth and young adults (vs. adults: adjusted odds ratio [AOR]=2.47, 95% CI: 1.71–3.58, *p*<0.001), men (vs. women: AOR=2.44, 95% CI: 1.64–3.64, *p*<0.001), and those with a mood and/or anxiety disorder (vs. those without: AOR=3.31, 95% CI: 2.27–4.82,* p*<0.001). 

***Vaping and former smoking***

As shown in Table 2, vaping and former smoking did not vary by age (*p*=0.09); however, over three-quarters of Canadians who reported vaping and former smoking were aged 25 years and older (78.6%). 

Vaping and former smoking varied significantly by sex (*p*<0.01) and gender (*p*<0.01), with higher prevalence among males (1.5%, vs. females, 0.9%), and men (1.5%, vs. women, 0.9%). Most Canadians who vaped and formerly smoked were males (61.1%) and identified as men (61.7%). 

Findings examining vaping and former smoking by visible minority status and Aboriginal identity were not reportable nor releasable due to data reporting requirements. 

Vaping and former smoking varied significantly by country of birth (p<0.001); those born in Canada (1.4%) had a significantly higher prevalence of vaping and former smoking than those born outside Canada (0.7%†). Most Canadians who reported vaping and former smoking were born in Canada (84.9%). 

Vaping and former smoking also varied by provincial region (*p*<0.001); prevalence was significantly higher in the eastern provinces (1.6%†), the western provinces (1.4%) and Quebec (1.7%†), as compared to Ontario (0.7%). The largest proportion of Canadians who reported vaping and former smoking lived in the western provinces (36.6%), followed by Quebec (33.1%), Ontario (21.8%) and the eastern provinces (8.6%†).

Vaping and former smoking did not vary by official language (*p*=0.07); however, most Canadians who reported vaping and former smoking (70.0%) indicated English as their first official language. 

No significant differences were observed in vaping and former smoking by education (*p*=0.15). More than half of Canadians who vape and formerly smoked had a postsecondary certificate/diploma or university degree or more (55.9%), almost one-third had secondary school education but no postsecondary education (30.3%), and the remainder had less than secondary school graduation (13.8%†). 

Vaping and former smoking did not vary by household income (*p*=0.52). Among Canadians who reported vaping and former smoking, household income showed a slightly skewed distribution, with a smaller share of respondents in the upper quantile. 

Significant differences were observed in vaping and former smoking by mental health (*p*<0.001); the prevalence of vaping and former smoking was significantly higher among those with a mood and/or anxiety disorder (2.3%), compared to those without such disorders (1.0%); however, the majority of Canadians who vape and formerly smoked had neither a mood nor an anxiety disorder (71.8%). 

The final multivariable model examining vaping and former smoking was fitted with age group, gender, provincial region and mental health. Results showed higher odds of vaping and former smoking among men (vs. women: AOR=1.81, 95% CI: 1.33–2.45, *p*<0.001); those living in Quebec (vs. Ontario: AOR=2.84, 95% CI: 1.75–4.62, *p*<0.001), the eastern provinces (vs. Ontario: AOR=2.37, 95% CI: 1.42–3.94, *p*<0.01), and the western provinces (vs. Ontario: AOR=2.14, 95% CI: 1.41–3.23, *p*<0.001), and those with a mood and/or anxiety disorder (vs. those without: AOR=2.55, 95% CI: 1.82–3.56, *p*<0.001). 

***Vaping and never/nonsmoking***

As shown inTable 2, vaping and never/nonsmoking varied significantly by age (*p*<0.001), with higher prevalence among youth and young adults (6.3%), compared to adults (0.3%†). Youth and young adults (75.7%) also represented most Canadians reporting this outcome. 

Vaping and never/nonsmoking also varied significantly by sex (*p*<0.001) and gender (*p*<0.001). Prevalence was higher among males (1.5%, vs. females, 0.8%), and men (1.5%, vs. women, 0.8%). Most Canadians who vaped and were never/nonsmokers were males (65.7%) and identified as men (66.0%). 

Vaping and never/nonsmoking did not vary by visible minority status (*p*=0.12); however, most Canadians who reported this outcome did not identify as a visible minority (83.7%). 

Findings examining vaping and never/nonsmoking by Aboriginal identity and country of birth were not reportable or releasable due to data reporting requirements. 

No significant differences were observed in vaping and never/nonsmoking by provincial region (*p*=0.52). Most Canadians who reported this outcome lived in the western provinces (35.9%) or Ontario (33.7%). 

Vaping and never/nonsmoking did not vary by official language (*p*=0.92); however, just over three-quarters of Canadians who reported this outcome indicated English (77.6%) as their first official language. 

Significant differences were observed in vaping and never/nonsmoking by education (*p*<0.001); Canadians with a postsecondary certificate/diploma or a university degree or more (0.6%†) had a significantly lower prevalence of vaping and never/nonsmoking, compared to those with less than secondary school graduation (2.5%), and those with secondary school graduation but no postsecondary education (2.0%†). Level of education was variably distributed among Canadians who reported vaping and never/nonsmoking: 38.4% had a secondary school graduation but no postsecondary education, 35.4% had a postsecondary certificate or diploma or a university degree or more, and 26.3% had less than secondary school graduation.

Vaping and never/nonsmoking varied significantly by household income (*p*=0.02): prevalence was significantly higher among Canadians in the upper household income quantile (1.6%), compared to the lower quantile (0.8%†). Among Canadians who reported this outcome, household income showed a skewed distribution, with a greater share of respondents in the upper quantile. 

No significant differences were observed in vaping and never/nonsmoking by mental health (*p*=0.13). Among Canadians who reported this outcome, 80.8% did not have a mood and/or anxiety disorder. 

The final multivariable model examining vaping and never/nonsmoking was fitted with age group, gender and visible minority status. Results showed higher odds of vaping and never/nonsmoking among youth and young adults (vs. adults: AOR=22.62, 95% CI: 14.06–36.39, *p*<0.001); men (vs. women: AOR=1.76, 95% CI: 1.21–2.56, *p*<0.01); and among those not identifying as a visible minority (vs. those identifying as a visible minority: AOR=2.31, 95% CI: 1.39–3.85, *p*<0.01). 

†Moderate sampling variability; interpret with caution.

## Discussion

To our knowledge, the study findings present one of the first in-depth, nationally representative characterizations of Canadians who vape, stratified by smoking status. 

While previous analyses of vaping correlates among Canadians aged 15 years and older showed no association with sex,^3^,^5^ our multivariable analysis yielded significant gender associations, with men having greater odds compared to women for each of the vaping outcomes stratified by smoking status. Previous research has identified an association between vaping and male sex among Canadian students.^4^,^6^ Results from the 2021/22 CSTADS^1^ showed differences in vaping by gender, with higher rates of vaping among girls/women compared to boys/men. While vaping prevalence was also high among students who identified as transgender, gender diverse and/or questioning, the contrast with boys/men did not reach statistical significance.^1^ Taken together, the findings suggest emerging trends in vaping by gender, which may be particularly important to monitor in young populations, given that they are substantially more likely to be nonbinary in gender identity and/or expressions.^20^

Dual use, as well as vaping and former smoking, were significantly associated with mood and/or anxiety disorders. These associations are perhaps unsurprising, given that these outcomes reflect present or past experiences with cigarette smoking, which is itself highly prevalent among individuals living with mental health issues.^10^ The common belief that smoking helps reduce stress and mental health symptoms or issues may cause concern that smoking cessation could worsen these outcomes. Evidence shows, however, that quitting smoking does not worsen and in fact may, in the long term, improve mood, mental health and abstinence from other substances.^21^,^22^

Evidence also shows that vaping products containing nicotine can help people quit smoking.^23^ Therefore, people who smoke should continue to be encouraged to quit, whether via vaping products or other forms of assistance. While an association between vaping and never/nonsmoking and mental health was not observed in the current analysis, research examining youth populations—the majority of whom do not have a history of smoking—suggests vaping is associated with poor well-being and greater delinquency,^24^ psychiatric comorbidities^25^ and lower perceived mental health.^1^ Thus, continued monitoring of mental health among individuals without a smoking history who vape, particularly youth, is warranted. 

Our results show that dual use was also significantly associated with young age (15–24 years). This finding is somewhat surprising, given that the rise in youth vaping between 2016/17 and 2018/19 was observed alongside continuing declines in cigarette smoking.^1^ However, this finding likely reflects the conflation of youth (15 to 19 years) and young adult (20 to 24years) respondents into a single category. While this was done to yield reportable results, it is important to note that young adults who vape are a distinct group with a prevalence trajectory that differs from both youth and older adults, and among whom the main reasons cited for vaping reflect a mixture of recreational use and use for smoking cessation.^2^

The outcome of vaping and former smoking also showed significant variation by provincial region. This result likely reflects the variation seen among Canadian provinces in the prevalence of former smoking, which has, over time, generally been lower in Ontario and higher in other regions, including in the western provinces and particularly in the eastern provinces and Quebec.^2^,^11^ As noted earlier, the impact of vaping products remains unclear for individuals who formerly smoked cigarettes;^7^ thus, more research is needed to understand the role these products play in smoking cessation and relapse, including longitudinal studies that examine motivations for use. 

The findings of this study deepen our understanding of the potential public health impacts of vaping products. First, the findings identify subpopulations with relatively higher and lower prevalence rates. For instance, vaping and never/nonsmoking was more prevalent among youth and young adults, men, and those who did not identify as a visible minority. Greater prevalence among young people is concerning, given that nicotine is an addictive substance, and exposure to nicotine during adolescence can harm the developing brain and may impact cognition.^26^,^27^ The findings may inform discussions regarding equity and research into effective communications and interventions for specific at-risk subpopulations, including primary prevention communications, such as media campaigns,^28^ as well as vaping cessation guidance.^29^

Second, study findings shed light on the composition of subpopulations that engage in these behaviours. For instance, analyses show that most Canadians reporting dual use were 25 years or older, identified as men, had higher levels of education and reported not having mental health issues. These results may further research into effective communications that encourage complete switching from cigarettes to vaping products.

***Strengths and limitations***

This study has several strengths, including the use of nationally representative data reporting on the use of vaping products across all Canadian provinces for the first time. In addition, examination of vaping by smoking status provides a nuanced understanding of this behaviour in terms of risk. Furthermore, the SGBA+ framework provides a rich and diverse lens through which to examine Canadians who vape.

However, there are also some limitations. To begin with, analyses were conducted using self-reported data, which may be subject to bias. Next, while the vaping question was sourced from the Tobacco Alternatives and Vaping (TAV) Rapid Response module of the CCHS, and thus aimed to assess the use of vaping products with or without nicotine, the question did not include a preamble to explicitly exclude cannabis; thus, it is possible that results may reflect vaping of various substances. In addition, while we acknowledge the limitations of interpreting past-30-day vaping as a measure of regular use,^30^ it is a commonly used measure and was the optimal measure available for analysis, given survey limitations. 

The use of the SGBA+ approach addresses individual-level factors; however, there may be interpersonal and societal factors related to vaping behaviour that were not addressed. Next, despite using a data source with a large sample size, we were limited in our ability to examine certain characteristics, such as sexual orientation and labour force activities, given reportability requirements, and disability status, which was not assessed in the 2020 cycle. Finally, data collection in the 2020 cycle occurred during the COVID-19 pandemic; data collection was interrupted between mid-March and September, which lowered response rates. Thus, results should be interpreted with caution, and continued monitoring of vaping among Canadians is warranted.

## Conclusion

The findings from our study allow for the identification of high-prevalence groups and deepen our understanding of who vapes in Canada, as a function of smoking status. The findings may inform further research in the areas of vaping prevention and cessation, including in relation to specific at-risk subpopulations, equity, and effective communications and interventions for specific audiences. 

## Funding

There was no funding for this research. 

## Conflicts of interest

None to declare.

## Authors’ contributions and statement

CDC, TM: conceptualization.

CG, DD, NF, GL, GW: formal analysis. 

CDC, TM, CG, DD, NF, GL, GW: interpretation of results. 

CDC: writing—original draft.

CDC, TM, CG, DD, NF, GL, GW: writing—review and editing.

The content and views expressed in this article are those of the authors and do not necessarily reflect those of the Government of Canada.
